# Enhancing LPG adoption in Ghana (ELAG): a factorial cluster-randomized controlled trial to Enhance LPG Adoption & Sustained use

**DOI:** 10.1186/s12889-018-5622-3

**Published:** 2018-06-04

**Authors:** Daniel Carrión, Rebecca Dwommoh, Theresa Tawiah, Oscar Agyei, Francis Agbokey, Miecks Twumasi, Mohammed Mujtaba, Darby Jack, Kwaku Poku Asante

**Affiliations:** 1 Department of Environmental Health Sciences, Mailman School of Public Health, Columbia University, 722 W. 168th Street – 11th Floor, New York, NY 10032 USA; 2 Kintampo Health Research Centre, Kintampo, Ghana

**Keywords:** Clean cookstoves, Household air pollution, Sustained use, Clean cookstove adoption, Behavioral intervention, Structural intervention, Biomass combustion

## Abstract

**Background:**

Three billion individuals worldwide rely on biomass fuel [dung, wood, crops] for cooking and heating. Further, health conditions resulting from household air pollution (HAP) are responsible for approximately 3.9 million premature deaths each year. Though transition away from traditional biomass stoves is projected curb the health effects of HAP by mitigating exposure, the benefits of newer clean cookstove technologies can only be fully realized if use of these new stoves is exclusive and sustained. However, the conditions under which individuals adopt and sustain use of clean cookstoves is not well understood.

**Methods:**

The Enhancing LPG Adoption in Ghana (ELAG) study is a cluster-randomized controlled trial employing a factorial intervention design. The first component is a behavior change intervention based on the Risks, Attitudes, Norms, Abilities, and Self-regulation (RANAS) model. This intervention seeks to align these five behavioral factors with clean cookstove adoption and sustained use. A second intervention is access-related and will improve LPG availability by offering a direct-delivery refueling service. These two interventions will be integrated via a factorial design whereby 27 communities are assigned to one of the following: the control arm, the educational intervention, the delivery, or a combined intervention. Intervention allocation is determined by a covariate-constrained randomization approach. After intervention, approximately 900 households’ individual fuel use is tracked for 12 months via iButton stove use monitors. Analysis will include hierarchical linear models used to compare intervention households’ fuel use to control households.

**Discussion:**

Literature to-date demonstrates that recipients of improved cookstoves rarely completely adopt the new technology. Instead, they often practice partial adoption (fuel stacking). Consequently, interventions are needed to influence adoption patterns and simultaneously to understand drivers of fuel adoption. Ensuring uptake, adoption, and sustained use of improved cookstove technologies can then lead to HAP-reductions and consequent improvements in public health.

**Trial registration:**

NCT03352830 (November 24, 2017).

**Electronic supplementary material:**

The online version of this article (10.1186/s12889-018-5622-3) contains supplementary material, which is available to authorized users.

## Background

Three billion people rely on biomass fuels for their cooking and heating needs worldwide. Biomass fuels consist of dung, wood, charcoal, crop waste, etc. [1]. Combustion of these fuels leads to high levels of particulate matter, carbon monoxide, polycyclic aromatic hydrocarbons, and other deleterious air pollutants [2–6]. In this context, these air pollutants are collectively referred to as household air pollution (HAP). The health effects of HAP are vast and wide-ranging. In fact, it is estimated that 3.9 million premature deaths are attributable to HAP annually [3]. Deaths attributable to HAP occur from a diverse set of diseases such as stroke, ischemic heart disease, pneumonia, cataracts, etc. [3, 7]. Although disease etiology and causal mechanisms are outstanding, it is widely acknowledged that HAP is a severe health threat. Therefore, efforts are being made to characterize and mitigate exposure. This protocol paper outlines the Enhancing LPG Adoption in Ghana (ELAG) study, a cluster-randomized controlled trial designed to increase LPG stove adoption and sustained use.

### Mitigating HAP exposures

HAP exposures result from combustion of biomass and other solid fuels in traditional cookstoves [8]. Public health proponents have looked to cleaner cookstoves to reduce HAP exposures [9, 10]. However, a number of recent studies establish that clean cookstoves do not automatically reduce HAP exposures [11]. Stove stacking, wherein households partially adopt the new technology while maintaining use of traditional cooking technologies, is a core challenge [12–15]. While partial adoption of cleaner cookstoves may partially reduce exposure, prior work has shown that these reductions are not sufficient to eliminate risk [16]. Another challenge facing clean cookstove interventions is that community-level emissions may substantially contribute to individuals’ exposure [17, 18]. Therefore, large scale adoption of clean cooking technologies may be required to decrease overall HAP exposures. These challenges have led to a body of scientific literature dedicated to clean cookstove adoption that addresses the determinants of uptake of the new technologies [19–22]. Ultimately these studies seek to understand opportunities to intervene on HAP exposure. For our purposes, ‘improved cookstoves’ refer to those which still utilize biomass, but increase the efficiency of combustion and thereby reduce HAP exposures. ‘Clean cookstoves’ on the other hand, refers to non-biomass stoves, including liquefied petroleum gas (LPG), induction, solar, biogas, etc.

### Efficient cookstove adoption

The scientific literature regarding cookstove adoption has identified numerous pre-conditions often associated with adoption. These variables can be broadly divided into three categories: household/setting characteristics, infrastructure, and knowledge and perceptions [20–22]. Household/setting characteristics are features that describe the household, their neighbors, and/or communities. Examples include ethnicity, religion, maternal and paternal education, female head of household, parental and family wealth/income, household size, and age. While it is regularly true that these variables predict adoption, the direction of the association varies across studies [21]. The reasons behind these inconsistencies are unexplained, but may represent uncontrolled confounding based on underlying constructs that are contextually relevant, or they may indicate the central role of local conditions in shaping decision processes.

Access-related factors associated with cookstove adoption include 1) financial, tax, and subsidy aspects (2) market development (3) regulation, legislation, and standards, and (4) programmatic and policy mechanisms [20–22]. Broadly defined, the access factors outline contextual physical and/or organizational facilitators of clean cookstove adoption and sustained use. Fuel access factors are also oftentimes specific to the stove type. For example, improved biomass cookstoves necessitate a different fuel infrastructure than LPG stoves. After an initial stove purchase, some stoves require repeated purchase of fuels. Users are then responsive to the price of the physical stove, but also fuel prices. Considering the fuel access environment of cookstove adoption is imperative for HAP-related interventions.

Understanding knowledge and perceptions preventing behavior change is vital to any health-related intervention. Studies have shown numerous associations with cookstove adoption, including knowledge/perceptions of: the health impacts of HAP, safety benefits of new cookstoves, time-savings benefits, improved cleanliness of newer stoves, social norms, newer cookstove users within a social circle, and the cultural appropriateness of technologies [20, 21]. Generally speaking, knowledge is regarded as highly modifiable whereas attitudes can be more challenging to alter [23, 24]. Both elements, however, must be aligned with a new behavior in order to observe behavior change [25].

### Challenges in cookstove adoption research

Studying cookstove adoption is both conceptually and methodologically challenging. Disciplines involved span the social, environmental, and health sciences, utilizing quantitative, qualitative, and mixed methods [26–30]. The field has largely employed observational study designs to date. While these studies are quite informative, they may be vulnerable to selection bias because individuals opt into each group of the study by deciding whether or not to purchase a stove, sustain use, etc. There may be underlying characteristics that predict entry into each group, thus limiting the generalizability of findings. Controlled trials can address these limitations through the randomization and follow up of participants, but there are few studies utilizing these study designs [31, 32].

Most cookstove adoption studies have focused on initial adoption versus sustained use. [21, 33]. This is an important distinction because adoption studies have largely focused on the enablers and barriers of initial stove acquisition and/or the use of the technology early in its adoption [13]. However, there are many reasons to believe that behaviors change over time. For example, researchers have noted situations wherein new stove use is high upon acquisition, but decreases over time. There are plausible reasons why participants would decrease use. New stoves could break with consistent use, and without access or means of repair, participants would likely default to the traditional stove. It is also possible that a household’s financial circumstances change and use falters. Without clear plans to recover from these external stimuli, users would resume traditional stove use. While there is a small number of studies focused on sustained use, that amount is growing. This is because researchers increasingly recognize the importance and complexity of the issue [13, 34, 35].

Household/setting characteristics, infrastructure, and knowledge and perceptions are all highly contextual issues. Although adoption has been extensively studied around the world, much work remains. Sub-Saharan Africa has the largest proportion of individuals using biomass fuels for cooking, and is the only region globally where traditional biomass use is still growing [36]. Sustained use studies are small in number and limited geographically. To our knowledge, there have been few studies in sub-Saharan Africa, demonstrating a need for continued research in an important region.

The goal of this paper is to outline the study design for the ELAG Study. The objectives of ELAG is to assess the effectiveness of two interventions on facilitating sustained use of LPG. A cluster-randomized trial with a factorial design is being used to test the effectiveness of two distinct interventions: 1) a behavioral change intervention using the Risks, Attitudes, Norms, Abilities, and Self-Maintenance (RANAS) model and (2) an access intervention to modify the ease of refueling [37]. The factorial design also allows us to evaluate the interaction of these two interventions. We deliver these interventions to mothers and, when possible, their partners. Sustained use is measured with stove use monitors (SUMs). These monitors will be in place for 12 months after intervention delivery. Sustained use will be assessed by analyzing the effect of the interventions on stove use in the last 6 months of the study period. We believe that this study will offer novel insights into the predictors of sustained use, strategies that can be employed to increase use, and important policy actions that can reduce exposures to HAP and its health consequences.

## Methods/design

This study builds on an ongoing successful collaboration between Columbia University in the City of New York and the Kintampo Health Research Centre (KHRC). In fact, the study is an outgrowth of the Ghana Randomized Air Pollution and Health Study (GRAPHS), which was a 5-year cluster-randomized controlled trial assessing the impacts of a HAP intervention on low birthweight and pneumonia [38]. GRAPHS included one control, and two intervention arms. LPG stove users and improved cookstove (Biolite) users served as the interventions and the traditional 3-stone fire users were the control arm. Ethical considerations dictated that the control arm would receive clean cookstoves upon study completion since LPG stoves are believed to reduce HAP exposures the most substantially. Given remaining resources upon study completion, all participants in the control and Biolite arms were scheduled to receive LPG stoves at study closeout. This provided an opportunity to assess patterns of adoption and sustained use of LPG cookstoves among a large group of participants.

### Hypothesis

We hypothesize that households that receive both the behavioral and access interventions will demonstrate higher levels of sustained use in the last 6-months of the study period, compared to those in the no intervention group, see Table 1.

**Table 1:** Allocation of clusters by study arm (and number of households)

	No educational intervention	Educational Intervention	Totals
	No. of communities *(households)*	No. of communities *(households)*	
No agent delivery	7 (271)	7 (243)	14 (514)
Agent delivery	7 (241)	6 (224)	13 (465)
Totals	13 (492)	12 (451)	27 (979)

### Study setting

**Fig. 1:**
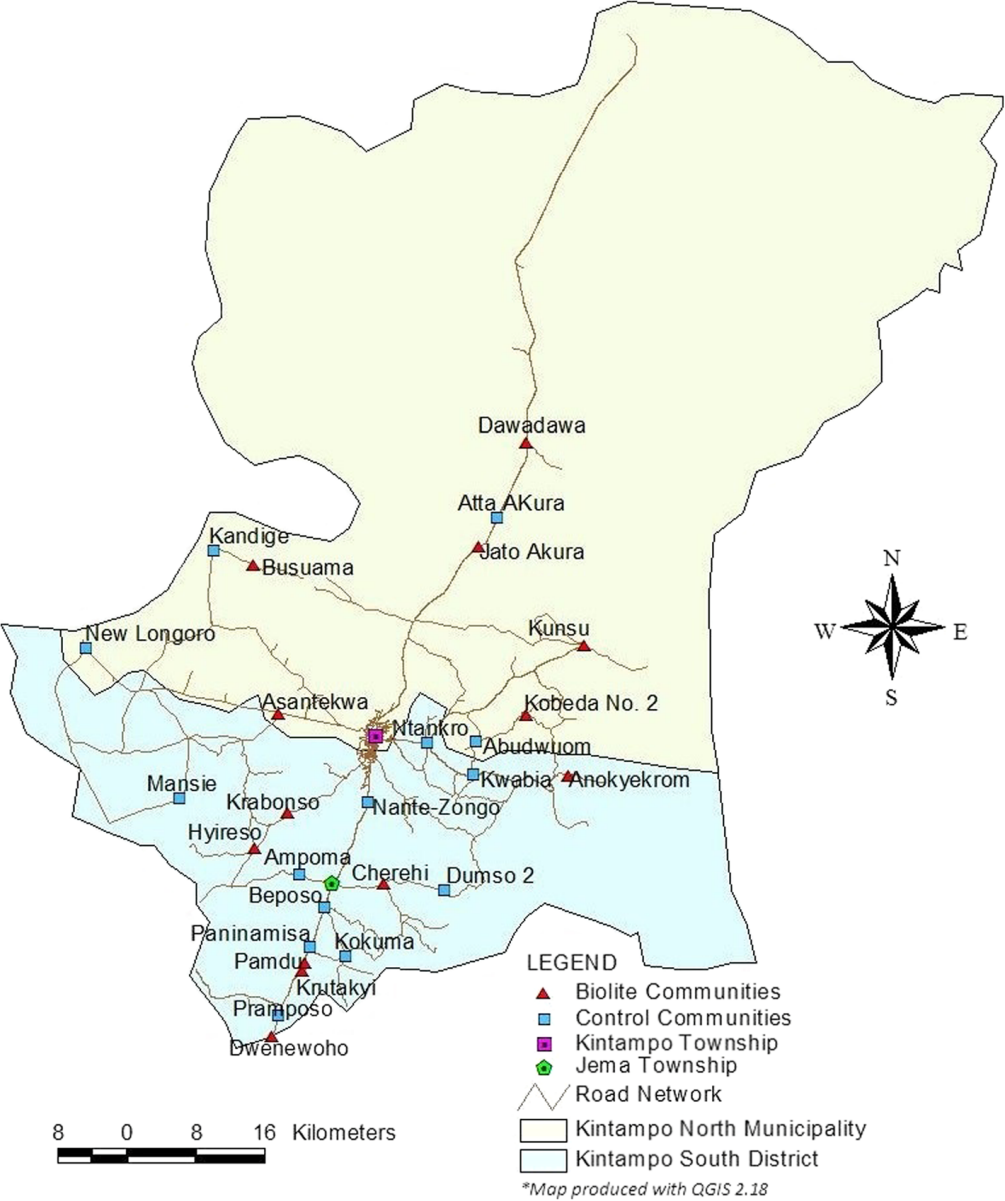
Map of Kintampo, the study area

The study area is in Kintampo North Municipality and Kintampo South District in the Brong-Ahafo Region of Ghana. This is a mostly rural area (population 176,480), see Fig. 1 [39]. Households in the study area traditionally use three-stone fires for their cooking needs. Ghana has a warm climate, with an annual average temperature is 26 °C [40]. Therefore, stoves are typically only used for cooking, not heating. There are two seasons, wet and dry. During the dry season most cooking takes place outdoors while enclosed or covered kitchen areas are the site of most cooking in the wet season. Wood is the main fuel source in the study area, but charcoal is used as well [41].

### Study eligibility & recruitment

ELAG participants are limited to participants who were enrolled in the original GRAPHS cohort and who: 1) were originally randomized to the Biolite or control arms of the study and 2) still reside in the KHRC study region (see Fig. 1). Participants are visited to assess interest in the new study, although, due to longstanding involvement with GRAPHS and other KHRC initiatives, we do not anticipate any issues reaching our recruitment goals.

### Ethics approval and consent

This study has received approval from the Institutional Review Board of Columbia University Medical Center and the Kintampo Heath Research Centre Institutional Ethics Committee. The study is registered with clinicaltrials.gov under NCT03352830. Informed consent is obtained by KHRC fieldworkers from all ELAG study participants prior to enrollment.

### Power

**Fig. 2:**
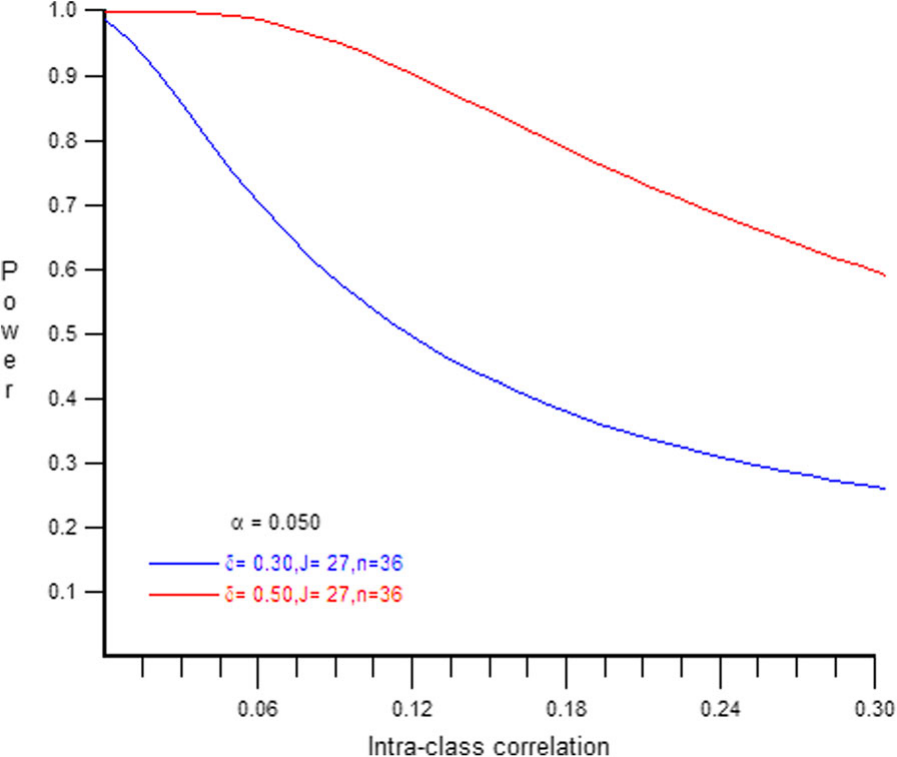
Power Calculations at .3 and .5 effect sizes

Power calculations reflect the cluster-randomized study design. Treatment is on the cluster level (2) while outcomes are on the household level (1). The outcome of the study is minutes of LPG use per day summed over a six-month period. Multilevel model power calculations with 27 clusters, 979 sample size and type I error at 5%. The mean for the reference group is estimated at 3000 min over 6 months, with equal group sizes. This is believed to be a conservative estimate based on our unpublished research in the region, which found ~ 3100 mean minutes of use over 20 weeks [42]. The effect size (Cohen’s D) is calculated with 1000 min as the pooled standard deviation, which is an overestimation based on our research. Multilevel power formulas were derived from Scherbaum, 2009 [43]. Intraclass Correlation Coefficients are unknowable given the novelty of the research, but several possibilities were modeled - visualized with the Optimal Design Plus Empirical Evidence version 3.1 software, see Fig. 2.

### Health promotion intervention

The study consists of two types of interventions, a behavioral change intervention and an access intervention. Our team selected the Risks, Attitudes, Norms, Abillities, and Self-Maintenance (RANAS) model to design a clean cookstove behavioral change intervention [37]. The RANAS model was originally designed and employed for issues pertaining to water, sanitation, and hygiene. However, we recognized its potential application for clean cookstove adoption. The core assumption that underpins the RANAS model is that each of these five behavioral factors are necessary, but not sufficient, to induce behavior change. After baseline data collection, ELAG households will be convened in cluster-wide meetings for LPG stove distribution and the behavioral change intervention. A research team member and a peer-adopter will collaborate to deliver the intervention. The peer-adopter is a participant from a GRAPHS LPG community who has maintained use of LPG after study conclusion. ELAG will rigorously employ RANAS to promote clean cookstove adoption, see Additional file 1 for the scripts used to guide the interventions.

Like other behavioral change campaigns, RANAS involves communicating risks associated with the traditional behavior. Not only should participants be aware of the severity of risks, but they should be made to recognize that they are vulnerable to those potential health outcomes [25, 44, 45]. Participants are first introduced to the concept of HAP. Then they are presented with a series of pictures that show adverse outcomes that have been shown to be caused by HAP, and that are recognizable to community members (cataracts, low birth weight, and respiratory diseases) [3, 7]. Additionally, pictures of blackened kitchen walls are used to explain that the same pollutants which dirty the walls also enter human lungs when exposed to smoke.

Attitudinal factors include perceptions of time, money, and effort associated with the behavior change, and the benefits of the new behavior. Following the presentation of risks, participants will be informed of the potential health benefits of reduced HAP exposure. Other benefits will be presented, such as reduced time dedicated to wood collection which can then be reallocated for educational or economic goals [46].

Normative factors describe perceived expectations from peers, leaders, and/or of one’s self. At this point the LPG peer adopter provides a testimonial regarding their experiences using LPG and overall appreciation of the technology. This is complimented with the a research staff explaining the Ghanaian government’s efforts to reduce HAP-exposures and protect Ghanaian natural resources from deforestation [47]. The community is then prompted to make a collective commitment to using LPG.

The ability-related behavioral factor represents the participants’ confidence in performing the new behavior. These abilities include safe, effective, and culturally-appropriate use of an LPG stove, which may require some orientation. Indeed there is some literature that documents new user’s reticence to use a new stove because it is unclear how culturally suitable it is [48, 49]. Therefore, it is important to demonstrate how to cook traditional Ghanaian meals on the LPG stoves. An LPG peer adopter will have a food demonstration where they cook a traditional meal for the participants in that cluster. On a longer timeframe, individuals may be unsure how to sustain the repeated payments required to refill the LPG cylinder [48, 50]. The intervention will also include a financial literacy orientation, which will provide strategies on how to smooth payments over time, including savings strategies and credit. A key component of the orientation will be a discussion among participants in smaller groups, on different scenarios of household financial limitations and the different strategies they would use to meet the financial obligation of using LPG stoves. Participants will also be provided with *susu* boxes (savings boxes) and encouraged to make weekly deposits towards the refilling of LPG cylinders.

Self-regulation factors provide continued orientation to the desired behavior in anticipation of conflicts or distracting cues to the old behavior. The self-regulation factor is designed with the assumption that relapse to the old behavior is inevitable for many individuals because outside circumstances cannot be controlled. ELAG addresses this factor by contracting and training Community-based Surveillance Volunteers (CBSVs) who will visit participants weekly to discuss the potential barriers to sustained use and brainstorm possible solutions. This is to assist sustained use efforts throughout the study period. CBSVs are trained to recognize and assist with issues that would interfere with LPG use, and serving overall as a resource for households. CBSVs have a long-standing presence in the communities, charged with tracking health and demographics in each community.

### Access intervention

Policy makers and researchers have suggested the importance of the ‘last mile’ (or, more realistically in rural Ghana, last 30 km [42]) of LPG delivery/accessibility, but few have empirically demonstrated the degree to which logistical barriers impact user demand [51–53]. This intervention will determine the degree to which physical accessibility of a product influences sustained use. Each community has at least one taxi driver or motor-king (tricycle cart) rider who can be paid to transport individuals or goods. KHRC will contract such drivers/riders in each community to provide pickup and drop off services for LPG cylinders. For this study, households in access intervention clusters will bear the cost of the refilling while the cost of transportation will be paid for with vouchers provided by the study. This intervention addresses an important gap in our knowledge surrounding sustained demand and use patterns of LPG fuels.

### Factorial design

Each cell of the factorial design will be functionally treated as an ‘arm’ of the study, see Table 1. This means that no one group will experience exactly the same set of conditions. The ‘No educational intervention’ and ‘No agent delivery’ will serve as the control group, while all other permutations of the two interventions will be compared to the control group during the analysis phase. As per the parent study (GRAPHS), this is a cluster randomized control trial. Randomization will occur on level 2 (villages) while outcomes will be measured at level 1 (households).

### Randomization

The parent study utilized a cluster-randomized design on coarsened exact matches to assign study treatments [54]. This study re-randomized clusters employing a covariate-constrained randomization approach with several identified prognostic covariates [55, 56]. The covariate-constrained randomization approach is a powerful allocation technique to ensure balance between arms in cluster randomized trials. Baseline covariates were chosen based on prior literature and theoretical relationships (see Table 2). When covariates are continuous, balance is determined via mean differences between treatment arms. Maximum permissible imbalance is designated a priori. Given these parameters, allocation amongst arms is randomly designated if below the maximum permissible imbalance. An independent epidemiologist performed the final randomization using the *ccrand* procedure in Stata [57]. Allocation was not revealed to field staff until after baseline data collection was complete.

**Table 2:** Covariate constrained randomization variables

Variable	Rationale
Community Asset Index	Scientific articles have shown that differential access to resources can be predictive in the uptake of new cookstove technologies (19–21).
Average Household Size	Scientific articles have shown that household size can be predictive in the uptake of new cookstove technologies (19–21).
Distance to Refueling Station	Study communities are scattered throughout the region at varying distances from the refueling center. Further distances are likely a deterrent to refuel for non-Agent delivery households, *see map*.
Households per cluster	To ensure roughly equal number of participating households per arm.

### Stopping guidelines

#### Positive stopping rule

Due to the nature of the intervention, the outlined study does not have a positive stopping rule. The hypothesis is that an educational intervention and an agent-delivery system will meaningfully increase sustained use of the clean cookstoves. Identifying the significance and magnitude of that improvement is a policy-relevant endeavor as it affords the opportunity to conduct cost-benefit analyses of expanded implementation. Our current outcome measure is time-dependent, wherein we will compare cookstove usage over the 12 months of the intervention.

#### Negative stopping rule

This is a behavioral intervention designed to determine effective strategies to increase the uptake of clean energies. The control group is a ‘business-as-usual’ approach, and we hypothesize the intervention groups will improve their uptake of the new technologies. We plan to halt study activities if 1) there are any LPG cookstove-related accidents that occur within our cohort and the study timeframe that result in permanent bodily injury or death, or (2) if the intervention groups show statistically significant decreases in LPG cookstove usage.

### Baseline data collection and covariates

Information on several constructs will be collected before delivering the behavioral and infrastructural interventions. Beyond standard baseline demographic and socioeconomic status surveys, a pre/post-test of RANAS model behavioral factors will be administered in order to document any changes in participants knowledge, perceptions, or attitudes regarding HAP and/or LPG stoves [25, 44, 45]. This will provide vital insight on the role of household/setting characteristics on sustained use, and the effectiveness of the various components of the behavioral intervention on knowledge/perceptions of HAP and cookstoves.

Many studies have shown that gender can play an important role in predicting cookstove adoption [58, 59]. Intra-household cooperation is a conceptual framework that makes explicit the bargaining process of decision-making within a household. Contrary to earlier economic theory, households do not operate as a unit. Instead, several actors negotiate decision-making, and power is rarely symmetrically distributed within the household [60, 61]. This asymmetry tends to follow gendered power dynamics. The concept of intra-household bargaining could thus mediate the relationship between the intervention and increased sustained use. Our study will utilize a modified version of the dictator game to assess intra-household cooperation [62, 63]. The game requires each partner of the household be separated temporarily for administration. Once separate, they are asked to select one of three envelopes with different pre-portioned sums of money. They must then decide whether to keep the entire sum of money for themselves, or direct all or a portion of it to their spouse and/or charity. The player can chose to send a certain amount of money to the third party. The money has a 50/50 chance of reaching that party and, if so, being doubled. In a perfectly cooperative household, it is within each individual’s interest to send all of the money to their partner. Intra-household cooperation, then, is measured as the ratio of money sent between partners over the entire amount received. Note that payout is hypothetical and currency will not actually exchange hands.

LPG fuel prices are a fluctuating covariate of interest, representing a potential access barrier to sustained use. Fuel wood is often free to the household because it simply requires that a family member spend time to collect material in the local environs or farms. Increasing LPG prices may lead some families to default to wood use [20, 21]. The research team will track fuel prices by calling or visiting the LPG refueling station on a regular basis. This will allow us to assess individual responsiveness to a dynamic fuel landscape.

### Outcome measures

The principal outcome of interest is minutes of LPG stove use over the last 6 months of the study. This time period is of interest in order to assess the effectiveness of the intervention on sustained use rather than initial adoption. Stove use will be measured via stove use monitors (SUMs). Each stove will be equipped with SUMs, which are iButton temperature loggers programmed to collect temperature data at 10 minute intervals [64]. This leaves the memory at capacity after 2 weeks. Field staff will visit households every 2 weeks to download the data. Monitors will be used to determine minutes of stove use during that period. Biweekly visits will also serve as a quality control measure because staff can address faulty readings, typically by replacing the iButton.

**Fig. 3:**
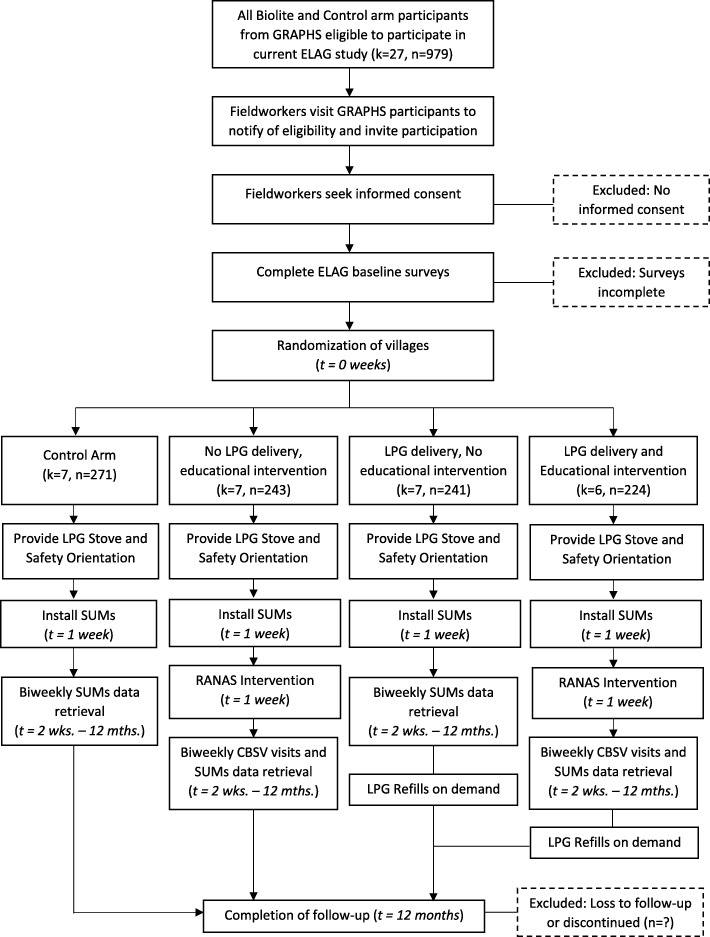
ELAG Study Flowchart

A secondary outcome of interest is stove use measured via weighing of LPG cylinders with a scale. Field staff are scheduled to visit households every 2 weeks to download SUMS data. They will use this opportunity to administer an LPG stove use questionnaire and weigh cylinders with a scale during their bi-weekly visits. Weighing cylinders is a cheaper alternative to measuring stove use, but it is possible that the measurements are biased. When a cylinder is weighed, participants will be asked if they refilled the cylinder within the 2 weeks and, if so, how much. It is possible that social-desirability bias is introduced in these measurements because participants are aware of our study interests and may misreport accordingly [65]. Determining the degree of bias or measurement error by comparison to SUMS data may be useful for future studies requiring stove use measurements in Ghana or similar contexts. See Fig. 3 for a study timeline.

### Data management

Field staff will administer numerous paper based surveys, and survey forms will be checked for completeness and consistency by a field supervisor. Data entry will be performed in the KHRC computer center. KHRC has well-established procedures for digitizing data and storing results in a relational database. The original paper records will be securely stored in the KHRC Data Center. Once paper records are transcribed, data will be anonymized for privacy protection. Personally identifiable information will be removed and a proprietary KHRC participant ID will link records. Clinical health information is not included in or collected by this particular study. Outside of KHRC, the only individuals permitted to access the data are those listed in the Columbia University IRB protocol. Standard KHRC and Columbia University procedures will be followed otherwise. Monthly reports providing summaries of enrollment and various field activities are discussed among the study team.

### Analysis

The primary analysis will be to examine the effect of the intervention on average stove use over the final 6 months of the observation period. Due to the nested nature of the observations, these data will be analyzed with a hierarchical linear model. The three intervention arms, consisting of the two interventions alone and one combined, will be compared to the control arm, which receives no intervention.

Several secondary analyses are also of interest. First, while the primary analysis is unadjusted for potential covariates, a secondary analysis will include covariates such as financial literacy, the number of unanticipated life events necessitating additional financial resources, ethnicity, intra-household cooperation, and household asset index. Second, additional analyses will look at the various trends across time, assessing stove use in the first 5 months of the study and over the 10 months. A third analysis will analyze sustained use across arms by demographic characteristics identified in improved cookstove adoption related systematic reviews. Finally, given that the Ghanaian government is already distributing LPG cookstoves and interested in increasing sustained use, this research is policy-relevant. Qualitative data will be collected and analyzed among key stakeholders involved in the intervention. This analysis can then be used to consider meaningful ways to scale the intervention based on existing fuel infrastructure.

## Discussion

ELAG represents a unique opportunity to understand various facets of LPG adoption and sustained use, principally the role of two distinct interventions to influence ongoing use, one via behavioral change promotion and another through fuel-access modifications. However, the large sample size, randomized design, and established cohort, allows for exploration of numerous other pivotal relationships. For example, assessing the role of gender via intra-household cooperation as a potential mediator of increasing sustained use is both innovative and instructive. Quantifying the degree to which unexpected life circumstances impedes sustained use is also novel and useful to policymakers.

Biomass cookstove use is a widespread source of air pollution, mostly in the developing world. However, much remains unknown regarding effective strategies to increase clean cookstove adoption and sustained use. Many studies to date have sought to demonstrate the health benefits of improved or clean cookstove, but have been largely unsuccessful, likely due to the difficulties of lowering exposures to health-relevant levels. Indeed, many studies have shown that exposure-reduction is a challenging endeavor [66]. Our study aims to document meaningful ways to increase adoption and sustained use, thereby reducing exposure. If successful, these strategies can then be scaled to measure the health impacts of exposure reductions.

## Additional file

Additional file 1: Script for delivering educational messages to Adoption Aim 3 Participants. (DOCX 26 kb)

## Abbreviations

CBSV: Community-based surveillance volunteer
ELAG: Encouraging LPG Adoption in Ghana study
GRAPHS: Ghana Randomized Air Pollution and Health Study
HAP: Household Air Pollution
KHRC: Kintampo Health Research Centre
LPG: Liquefied petroleum gas
RANAS: Risks, Attitudes, Norms, Abilities, and Self-regulation
SUMs: Stove use monitors
