# Supplementary material for: Enhancing LPG adoption in Ghana (ELAG): a factorial cluster-randomized controlled trial to Enhance LPG Adoption & Sustained use
Source: BMC Public Health. 2018 Jun 4;18:689. doi: 10.1186/s12889-018-5622-3 (PMC5987623; doi:10.1186/s12889-018-5622-3)
Supplement: Supplementary file 1 — Script for delivering educational messages to Adoption Aim 3 Participants. (DOCX 26 kb) [file 12889_2018_5622_MOESM1_ESM.docx]

**KINTAMPO HEALTH REASEARCH CENTRE (KHRC), GHANA**

**ADOPTION OF CLEAN COOKSTOVE STUDY: AIM 3**

**Script for delivering educational messages to Adoption Aim 3 Participants**

**RISKS:**

**Step 1:** Start by telling participants that:

*“I am going to tell you about some of the risks that you are exposed to when you engage in activities that result in household air pollution.*

**Step 2:** Deliver key message to participants by saying:

1. *“Household air pollution occurs when you burn fuels such as animal dung, charcoal and wood for cooking inside the home. These fuels produce a lot of smoke”*

**Step 3:** Display corresponding picture accompanying key message for participants to see by saying:

*“Please watch this picture and describe what you see”*

**Step 4:** Listen to participants’ comments on the picture displayed and discuss where necessary.

**Step 5:** Deliver the last two (2) key messages under risks and repeat steps 3 and 4 above after each message is delivered:

1. *“The smoke produced leaves people’s homes dirty as a result”.*
2. *“This is also what you and your family breathe in when you are cooking or near one of these stoves”*

Allow five (5) minutes for questions or contributions on the session on risks.

**VULNERABILITY:**

**Step 1:** start this session by telling participants that:

*“Now I am going to tell you about how vulnerable we are to contracting some diseases related to smoke”*

**Step 2:** Deliver first key message by saying:

1. *“Exposure to the smoke may lead to: Cataracts, Lung diseases, Pneumonia, Heart disease, Reduced birth weight”*

***NB: display corresponding picture of the health condition after mentioning it.***

**Step 3:** Deliver the next key message by telling participants how vulnerable we are in getting the diseases mentioned in step 1 above by saying:

1. *“You and I are vulnerable of getting any of these severe diseases if we continue to inhale the smoke.”*

Allow five (5) minutes for questions or contributions on the session on vulnerability.

**SEVERITY:**

**Step 1:** Start by telling participants that:

*“I am going to tell you about how severe the impact of these diseases can be on your quality of life.*

**Step 2:** Deliver key message to participants by saying:

*“These diseases can impact your quality of life very negatively:*

- Cataracts – not being able to see (properly) and therefore become less productive, burden to the household
- Lung diseases – can be deadly depending on severity. Can also impact productivity.
- Low birth weight – may make it harder for your child to be healthy in the long term.
  - This may also be costly for your time because of taking your child to healthcare facilities.”

**Step 3:** Display the picture of the skull *(which depicts death*) to show the extent of severity for participants to appreciate.

Allow five (5) minutes for questions or contributions on the session on risks.

**ATTITUDINAL FACTORS:**

**Step 1:** Start by telling participants that:

*“I am now going to tell you about some of the benefits of using LPG cookstove.”*

**Step 2:** Deliver key message to participants by saying:

*“Using LPG is very safe and healthy, hygienic and clean, cooks fast, utensils don’t get black, saves time and it is easy to clean”*

**Step 3:** Mention the specific benefits as stated below:

- Healthy – reduces the risk of lung diseases like pneumonia, heart diseases, and reduced birth weight.
- Safety – burns from traditional stoves, snake bites, cutlass and other injuries from firewood collection
- Time savings – you don’t need to collect wood and you cook faster.

Ask participants how long it takes them to get firewood:

*“How long does it take you to get firewood?”*

- Education/Economic opportunity – imagine what you could use your time for? Perhaps you and your children could spend more time on education or extra time working to make more money.
- You will be able to use your LPG anytime you want to cook

Allow five (5) minutes for questions or contributions on the session on risks.

**NORM FACTORS:**

Peer to give testimonial about safety, time savings, and education /economic opportunity.

**Step 1:** Tell participants that you are now going to invite someone who has adopted the LPG cookstove and is here to share her experiences with them.

**Step 2:** Invite the peer adopter to share her experience on the use of LPG cookstove with the participants.

Allow five (5) minutes for questions or contributions on the session on risks.

**OTHERS’ APPROVAL:**

**Step 1:** Tell participant about the approval of others such as the government through the Ministries, Agencies and Departments are interested in encouraging the use of LPG by saying:

*“The Ministry of Petroleum and Energy, Forestry Commission and the Ghana Health Service are all in support of people who use the LPG cookstove. The government of Ghana through the Ministry of Petroleum and Energy is distributing LPG cookstoves, cylinders and accessories to women in rural communities as way of encouraging many people to use LPG”.*

**Step 2:** display picture showing the public distribution of LPG cookstoves, cylinders and accessories to beneficiaries of the rural LPG Programme.

**Step 3:** Tell participants that:

*“The ministry of petroleum and energy, forestry commission and the Ghana health service all disapprove of the traditional cookstove because it pollutes the environment and depletes the forest.”*

**Step 4:** show picture of deforestation to the participants.

**PERSONAL IMPORTANCE:**

Step 1: Tell participants that:

*“People who are using the LPG cookstove are seen as modern and up-to-date because:*

1. *They spend less time in cooking*
2. *They cook in very clean cooking environments since no smoke/ashes are generated to make the cooking area dirty.*
3. *They cook very tasty foods devoid of smoke since no smoke is emitted to change the taste of food.*
4. *Their cooking utensils are always neat and require less time and effort to wash/clean them.*
5. *They are able to spend quality time to bond with their spouses and family.*

*They are able to cook anytime and in any weather.*

Step 2: show picture of a woman very relaxed whilst cooking on her LPG cookstove.

**ABILITY FACTOR:**

Step 1: demonstrate to participants how to use the LPG cookstove.

Step 2: group participants and let each member of take turns to try to rehearse how correctly use the LPG cookstove while you go round to assist those who have difficulty in doing it until they get it.

Step 3: Deliver key message to participants by saying:

“You should use your LPG every day and anytime you have to cook”

Allow five (5) minutes of questions and contributions from the participants.

**FINANCIAL EDUCATION:**

Step 1: Start by telling participants the purpose of the financial education

“*To discuss how participants can cope with the financial implication of using the clean cook stove to ensure continual use*”.

Step 2: Tell participants about household financial management:

“*Many people keep records of how much money they have, how much is spent and saved at the end of day, week, month and even year. This can be done for household revenue or a business venture.*

*Doing this helps to know how much money has been received, spent and saved. It also helps to prevent or minimize misusing money and also plan for future expenditure*”.

Step 3: Discuss money saving

Tell participants:

“*Saving is when you keep money apart and you do not spend it, unless you have planned to use it for some purpose*”.

Ask Participants:

“*People save for various reasons; what are some of your reasons for saving?”*

Allow for five (5) minutes of response from participants.

If any of the following is not mentioned by participants, you can mention it to them

- Household upkeep (feeding, health care, fuels for cooking, rent, utilities, etc. for the next month or the following year). **Facilitator should put more emphases on saving towards LPG purchase and parts replacement at this point**.
- To start or expand your business ( e.g. buy more farming tools, more seeds for the next farming season, buy hair dryers, – **Facilitator should** **tailor examples to fit participants’ occupation**…etc.)
- To prepare for emergency/unforeseen situations (health care: You child is sick and is admitted to the hospital; The school fees have suddenly been increased,
- For social events: (e.g. Funerals, weddings, naming ceremonies, etc.)
- Long term family goals (savings towards a house, a car, travel, etc.)
- Educational purposes (e.g. saving towards child/spouse/family future education – high school, tertiary, vocational, etc.)
- To improve your way of living (examples: Building a house, Sending the children to a good school
- To replace or repair old or damaged equipment and utensils; bowls, pots, spraying machine and cutlass (if a farmer), etc.

Step 4: Discuss mode of saving

MODE OF SAVING

Ask participants: *“In which ways have people been saving their money?”*

[Allow for five (5) minutes of response from participants on some of their personal reasons for saving]

Accept all answers, and proceed with the explanation

- Informal saving [e.g. personal or group susu]
- Saving with a financial institution [e.g. Banks, micro-finance, savings and loans institutions, etc.]
- Investment accounts [fixed deposit, treasury bills, shares, etc.]

Step 5: Group discussions

Put participants into groups and each should select a leader who will be responsible for presenting the groups issues. Each group should have a research team member who will be responsible for moderating discussions among the participants and notes taking.

**Discussion 1: case study**

SCENARIO 1: Read out the scenario to participants

*“Maame* **(use a name that is common in the community the discussion is being held***) is a Kenkey seller in Nante Zongo* **(use name of the community the discussion is being held)***. She makes GH¢50 income a week from her sales. She need GH¢20 to buy ingredients for her Kenkey each week. She has plans of expanding her business in the next year. She also wants to help her husband pay for their child’s school fees for the next academic term and also renovate their current house to include a kitchen and an additional room for their son. In addition to these, she also has to take care of her personal female needs, contribute to food for her household and also refill her LPG cylinder which will finish in two months’ time.”*

**Task for Participants: How do you think she can manage to do all these with her income?**

SCENARIO 2: Read out the scenario to participants

*“Nana* **(use a name that is common in the community the discussion is being held***) is the wife of Mr. Kwame* **(use a name that is common in the community the discussion is being held***). She does not have a regular job for which she earns regular income. She occasionally helps her husband with his farm. Her husband occasionally brings home food crops from his farm and gives her GH10 each week for housekeeping. Nana has personal female needs she has to take care of and also uses an LPG cylinder which has to be refilled every three months.”*

**Task for Participants: How do you think she can meet her needs?**

Allow for about 10 minutes discussion among participants within the groups and another 5 minutes for each group to share their thoughts with the bigger group.

Main issues:

- Scenario 1
  - Saving towards her needs including the LPG
  - Cut cost/Minimize spending on other things that she may want but not really be a need or necessity such as buying clothes, shoes, cooking utensils, etc. on a whim rather than need
  - She could work more to increase her sales (increase the quantity of kenkey cooked a day, sell for longer hours, sell other things in addition to her kenkey like sachet water, etc., find more market or consumers for her kenkey

- Scenario 2
  - Generate income: She could find a job – maybe sell few things e.g. sachet water, work on people’s farm or even her husband’s farm to increase production, or even identify something people could be interested in and sell or produce it,
  - She could cut down on cost
  - She could save bits of the money her husband gives her

**Discussion topic 2: Thinking through saving for LPG refilling**

In their smaller groups, participants should discuss the following topics with a research team member moderating the discussion:

- What resources do they need to be able to refill their LPG cylinders whenever they run out
- What are the possible steps they can take to generate the resources they need
- In which ways can they support each other in order to finance the use of clean cook stoves
- In which ways can they promote the use of clean cook stoves in their communities?

Step 6: closing discussion on financial education

Discuss the following with participants:

- Costs associated with using LPG
  - Cost of refill: [ GH₵70.00 for the 14.2 kg. however you can refill any quantity you have depending on how much money you have]
  - Cost of transportation: depending on the transportation cost in your community
  - Cost of accessories for replacement in case of damage [ranges from 2.00 for valve replacement, GH₵10.00 for tube replacement (depending on the length you want), GH₵30.00 for regulator replacement (depending on the brand), etc.

With all these, the highest cost is with the cost of refill. If you save GH₵1.00 a day, you can save about GH₵ 60.00 in two months which can be used to refill the cylinder.

You will be given a susu box in which you can save daily amounts of GH₵1.00 or less or even more if you can afford it. [Show participants the susu box and how it can be used]

Step 7: invite the LPG adopter to share her experience on how she (or household) copes with the financial implication of using LPG.

*****Do a cooking demonstration to conclude session*****

**CONFIDENCE IN CONTINUATION:**

Deliver the following key messages to participants on possible challenges they might face in the course of using the LPG cookstove and how to overcome them to ensure continuous use by saying:

1. *“You should contact your neighbours or CBSVs if you have any challenge with your stove”.* ***NB: Introduce the CBSV to participants to see.***
2. *“You may in the course of using the LPG cookstove encounter some difficulties/setbacks which may discourage you and make you regret your decision to use the stove. When any of these things happen, do not despair but treat them as teething problems/challenges that you/they can overcome. For challenges as:*
   1. Spoilt regulators, valves, leaking tubes and cylinders, consult the filling station attendant at any LPG filling station to get it checked and fixed or parts to be changed. *Alternatively, inform the study team at KHRC through the field supervisor for a possible replacement???*
   2. *Shortage of gas when cooking. Try as much as possible to avoid this by getting an additional cylinder filled with LPG to serve as backup to fall on in such situations.*

**OTHERS’ BEHAVIOUR: Public Commitment:**

Get participants to make a public pledge / commitment to use LPG for cooking by asking them the following question:

*“Which of you here is ready and willing to use the LPG cookstove?”*
